# The use of topical vaginal estrogens in postpartum women: A systematic review

**DOI:** 10.1111/aogs.70241

**Published:** 2026-05-04

**Authors:** Aysha Waheed, Annika Taithongchai, Dudley Robinson

**Affiliations:** ^1^ Department of Urogynecology King's College Hospital London UK

**Keywords:** pelvic floor, postpartum atrophy, postpartum pelvic floor dysfunction, topical estrogens, vaginal estrogens

## Abstract

**Introduction:**

Topical vaginal estrogens have been used in the management of postmenopausal vulvovaginal atrophy for some time, locally reintroducing exogenous estrogens to the estrogen‐receptive tissues of the female urogenital tract following a decline in systemic levels. The postpartum period is similarly a state of relative estrogen depletion due to the antagonistic effect from raised prolactin levels throughout pregnancy and lactation; this is thought to contribute to post‐delivery pelvic floor dysfunction and vaginal atrophy. Given the similar etiology, there may be a role for topical vaginal estrogens in postpartum women.

**Material and Methods:**

A systematic review was performed evaluating the use of topical vaginal estrogens in postpartum women. This was performed as per PRISMA guidelines, with articles screened from online databases from their inception to October 2025.

**Results:**

Three studies were identified that met the inclusion criteria, including a total of 85 women. Two studies focused on the molecular and cellular composition of tissues from postpartum women using topical vaginal estrogens, and one double‐blind randomized placebo‐controlled trial primarily evaluated postpartum atrophy in the presence and absence of vaginal estrogens. Tissues exposed to estrogen appeared to have increased cellular proliferation, while the Vulval Assessment Score (VuAS), a patient‐subjective validated scoring system for vaginal atrophy, was found to be lower in those using vaginal estrogen cream for 12 weeks. No statistically significant difference in bladder, bowel, or sexual function was noted. No serious adverse outcomes were reported, and the use of vaginal estrogens appeared acceptable to women.

**Conclusions:**

There is a paucity of data currently available regarding the use of topical vaginal estrogens in the post‐partum period. Although likely safe for use, further evidence is required to be able to make firm conclusions about their efficacy for postpartum vaginal atrophy, and further high‐quality trials are required in the future.

## Graphical abstract

A postpartum reduction in circulating estrogen levels, due to antagonism from elevated prolactin levels, further elevated during breastfeeding, can result in pelvic floor dysfunction, including bladder, bowel and sexual dysfunction, pain and implications for perineal wound healing. This systematic review aims to evaluate the current evidence of using topical vaginal estrogens to locally re‐introduce the hormone to the estrogen‐receptive tissues of the female urogenital tract during the postpartum period.

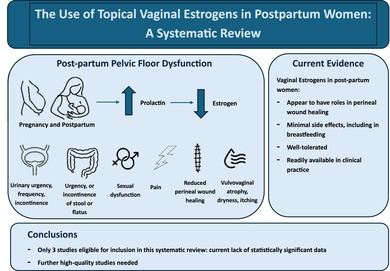

**Abbreviation**
VuAS: Vulval Assessment Score

**Key Message**

Reintroducing topical estrogens to estrogen‐deplete postpartum urogenital tract tissues may increase cellular proliferation and appear safe and acceptable to women. Current data are limited. More evidence is required regarding possible influences on pro‐inflammatory markers and objective improvement in pelvic floor dysfunction parameters.

## 1 INTRODUCTION

Estrogen receptors have been identified throughout the female urogenital tract and pelvic floor, including in the vagina, clitoris, bladder, internal and external anal sphincters, and the pelvic floor musculature.^1^, ^2^ Studies performed in estrogen‐deplete subjects (postmenopausal women or, often, oophorectomized rats) have demonstrated tissue changes including disorganized collagen deposition, fibrosis, and atrophied detrusor muscle. These findings improved with the re‐introduction of exogenous topical estrogens,^3^ suggesting not only the estrogen receptivity of these tissues but also an integral role for estrogen in maintaining structural integrity and potential roles in healing mechanisms, perhaps through promotion of cellular proliferation.^4^, ^5^ Vaginal estrogens, alone and in combination with other medications, have also been shown to improve symptoms of overactive bladder syndrome and stress urinary incontinence^6^, ^7^; however, other reviews and metanalyses have been unable to conclude a definitive benefit for their use in other pelvic floor disorders, such as pelvic organ prolapse,^8^ largely due to the heterogeneity of the available studies, not only in terms of outcome measure but also in the type and dose of vaginal estrogens used within the trials.

Similar to the postmenopausal state, the postpartum period is a state of relative estrogen depletion: the marked increase in systemic prolactin levels during pregnancy, and sustained throughout breastfeeding, results in an inhibitory effect on ovarian estrogen production.^9^ Although it can occur in all women, postpartum vaginal atrophy is found to be more common in lactating women.^10^, ^11^ This can result in vaginal pain, discomfort, and itching, as well as dyspareunia, and may impact on wound healing and bladder and bowel function.^1^, ^4^, ^12^, ^13^ Postpartum pelvic floor dysfunction is likely multifactorial, with perineal trauma and mechanical stressors also contributing to the symptoms experienced by women following a vaginal delivery.^9^

Postpartum pelvic floor dysfunction is common: 54% of women report stress urinary incontinence,^14^ 8% report urge urinary incontinence at one‐year postpartum^15^ and 29% report fecal incontinence within six months of delivery.^16^ Postpartum vaginal atrophy and dyspareunia is also common: 67.6% of breastfeeding women^10^ were found to have vulvovaginal atrophy, and 80% of breastfeeding women reported postpartum dyspareunia.^9^, ^17^ These levels were higher than those who were not breastfeeding, suggesting estrogen being a significant factor to these symptoms.

Despite the prevalence figures reported in the literature, there is a current paucity of data regarding the management of postpartum pelvic floor disorders, in comparison to the similar symptoms experienced by postmenopausal women. This may be due to a perception that such symptoms are part of a typical postpartum course, or for fear of embarrassment due to the often‐sensitive nature of pelvic floor dysfunction, preventing women from seeking advice or accessing services. In 2023, NHS England announced plans for a mandatory Perinatal Pelvic Health Service, enabling accessible pelvic health care for all women from delivery to at least one year postpartum^18^ highlighting the importance of meeting the needs of this patient population, where previously minimal guidance for both patients and clinicians existed. Guidelines currently exist for the management of postmenopausal vulvovaginal atrophy, with the National Institute of Health and Clinical Excellence (NICE) recommending the use of topical vaginal estrogens.^19^ Although anecdotally some clinicians utilize similar management for breastfeeding postpartum women, given a likely similar etiology, there is a current lack of evidence‐based guidance to help care for symptomatic postpartum women.

The aim of this systematic review is to evaluate the current evidence available for the clinical role of topical vaginal estrogens in postpartum women.

## 2 MATERIAL AND METHODS

### 2.1 Selection criteria

Studies were eligible to be included in this review if they recorded original research concerning the use of topical vaginal estrogens, in any form, be that a cream, pessary, or intravaginal ring in postpartum women, including the period from delivery to 12 months postnatal. Only human studies were included.

Exclusion criteria included studies which used systemic estrogen preparations, animal studies, and the inclusion of women outside of the postpartum period, including postmenopausal women. Unpublished data, review articles, meta‐analyses, stand‐alone abstracts, editorials, letters, and book‐chapters were excluded from this review, as were any reviews where the full text was not available in English.

### 2.2 Search strategy

A search from database inception to 30th October 2025 was made using PubMed, performed in accordance with the PRISMA guidelines. PROSPERO ID number is 1276519. The search term (Appendix S1) was developed to identify all relevant articles for possible inclusion into this review. Additional searches using the Embase and Medline databases were also carried out, as well as supplementary internet searches and citation tracking. Articles were initially screened by title and abstract according to the pre‐defined inclusion and exclusion criteria, followed by full‐text review of potentially eligible articles to confirm suitability for inclusion. Figure S2 outlines the literature search performed.

### 2.3 Data extraction

Following the identification of eligible studies, relevant data for inclusion into the review was extracted into a summary table by the first author. The data collected included demographic characteristics of the sample population, formulation of vaginal estrogen used, the outcome variables, study methods, and relevant results.

## 3 RESULTS

A total of three articles were found to meet the inclusion criteria for this review, which reported on a combined total of 85 individual subjects. Two prospective case‐controlled studies by Luz (*n* = 10) and Bochenska et al. (*n* = 16)^20^, ^21^ evaluated in vitro cellular, structural and molecular characteristics of postpartum vaginal tissue exposed to vaginal estrogens compared to controls. One double‐blinded randomized placebo‐controlled trial by Smith et al.^22^ (*n* = 59) studied the impact of vaginal estrogens on vulvovaginal symptoms in primiparous women who had sustained a perineal laceration following a term vaginal delivery. Two of the trials were pilot studies,^21^, ^22^ with the trial by Smith et al. underpowered to assess a change in Vulvar Assessment Score (VuAS) due to inadequate recruitment.^22^

All the participants in the included studies had undergone a vaginal delivery, although information regarding the gestation of delivery was only provided in one study.^22^ A third‐ or fourth‐degree perineal tear was sustained by 62% of the sample population in the study by Bochenska et al.^21^ and 12% of the population in the study by Smith et al.^22^ Luz stated that the women included in their sample were “normal postpartum women” with no formal definition or inclusion criteria given, nor any further demographic information of participants.^20^ The number of breastfeeding women included in the studies varied, from 100%^20^ to 75%^21^ to 46%,^22^ although of note each has differing sample sizes and trial durations (ranging from 22 days to six months postpartum) which may have impacted on the values given (Table S3). Although all the data from Luz et al. is taken from breastfeeding subjects, neither Bochenska et al. nor Smith et al. performed sub‐analyses on breastfeeding subjects.

Topical vaginal creams were used as the vaginal estrogen preparation in all three studies; however, the type of vaginal estrogen, duration of use, dose and administration differed between them. A placebo cream (Versabase) was used as a comparator in one study.^22^ A standardized dose and regimen for application was used in two studies,^20^, ^22^ although differing preparations were used: 5 cubic centimeters (cc) daily dose of 0.1 mg/cc dienestrol until 15 days postpartum^20^ and 0.01% estradiol cream 1 g twice weekly for 12 weeks.^22^ One study did not standardize for the dose or frequency of use of vaginal estradiol cream,^21^ with subjects applying the cream from between every night to twice a week. Only one adverse effect was noted in any of the included studies: one urinary tract infection (UTI) was reported by a participant in the placebo arm of the trial and was found to have been non‐serious and not directly related to the trial.^22^

Histopathological appearances of postpartum vaginal tissue, with and without the use of vaginal estrogens, were investigated in two studies: Luz et al. investigated postnatal vaginal fluid smears,^20^ while Bochenska et al. used samples of symptomatic perineal and vaginal granulation tissue excised from postpartum women.^21^ Both studies identified an increase in markers of cellular proliferation in the samples exposed to vaginal estrogens. Luz demonstrated that proliferative cell types were present in vaginal fluid as early as three to four days postpartum, with strong proliferation seen on days 11–15 in the estrogenized samples. This was primarily attributed to increased cornification and karyopyknosis, intracellular activity occurring following apoptosis in preparation for cellular regeneration.^20^ Following the termination of vaginal estrogen usage, these processes reduced by approximately 40% and 50% respectively. No comparative data is available for any changes seen in control samples over this time period. In the study by Bochenska et al., no cellular structural changes were observed between the granulation tissue samples from participants using vaginal estrogens compared to those who had not. CCND1, a cell‐cycle regulator involved in cellular proliferation, was found to be increased in estrogenized samples, but not to statistically significant relevance (*p* = 0.07). Conversely, Ki67, another cellular proliferation marker, showed no increase compared to controls.^21^

Two trials studied the influence vaginal estrogens may have on inflammatory markers.^20^, ^21^ Whereas Luz found a reduction in inflammatory cells (unspecified) in estrogenized vaginal smear samples after day four postpartum,^20^ Bochenska et al. found no difference in the expression of the pro‐inflammatory cytokines TNF‐α and IL‐11 in the vaginal granulation tissue between the two groups.^21^

Only one study investigated estrogen receptor expression in the presence of topical vaginal estrogens. Immunohistochemical staining to identify estrogen receptors found no difference between the two trial arms in estrogen receptors 1 and 2 (ESR1 and ESR2).^21^ In both groups, the alpha estrogen receptor (ERα) was more abundant than the beta receptor (Erβ), but more so in the estrogenized sample, with levels found to be 111 times that found in the control group.

One study explored the role of vaginal estrogens on elements of pelvic floor dysfunction using validated questionnaires to give quantitative results, including the Urinary Distress Inventory‐6 (UDI‐6) for urinary symptoms, the Fecal Incontinence Severity Index (FISI) for bowel and fecal incontinence symptoms, and the Female Sexual Function Index (FSFI) for sexual dysfunction.^22^ No statistically significant difference was observed between the estradiol and placebo groups for any of these variables. The Edinburgh Postnatal Depression Scale (EPDS) was also included, and again no statistically significant difference was observed between the two groups. The validated Vulvar Assessment Scale (VuAS) and Vaginal Health Assessment (VHA) were additionally used to assess subjective and objective vaginal characteristics. The VuAS is a 4‐item patient‐reported scoring system assessing vaginal dryness, soreness, irritation, and pain, each from mild (1 point) to severe (3 points). It was initially developed for use in cancer patients. The VHA is a clinician‐performed examination of the vagina, including commenting on vaginal moisture, elasticity, and the presence of ruggae. At the 12‐week follow‐up, the mean VuAS score was 0.20 in the placebo compared with 0.10 in the estradiol arm, resulting in an absolute difference of −0.10 (90% CI −0.20 to 0.01). No difference was seen at the six‐week or six‐month follow‐ups.

One trial explored the acceptability of using vaginal estrogens and their subjective efficacy.^22^ This was performed using a qualitative survey of the double‐blind participants. Overall, 52% of subjects in the estradiol treatment arm reported being “very satisfied” with their treatment and 0% were “somewhat dissatisfied.” Participants mostly found that their symptoms were “very much better” (52%), and that the estradiol cream was “easy” or “very easy” to use (89%). Good compliance was demonstrated, with 96% reporting using the cream “most of the time” or “all of the time” as per the prescription of twice weekly application. In comparison, only 33% of the placebo arm subjects reported being “very satisfied” with their allocated treatment, with the majority (41%) reporting being “neutral/unsure,” and fewer (31%) reporting symptoms being “very much better” at the end of the study. Compliance was also reduced in the placebo group, with only 66% using their treatment as prescribed “all of the time” or “most of the time.”^22^

## 4 DISCUSSION

This systematic review summarizes the available evidence regarding the use of topical vaginal estrogens in postpartum women. The heterogeneity of the outcomes studied in the three included trials makes deriving meaningful comparisons between the data challenging. Sample sizes were small in all trials and the historic nature of the study by Luz, using less rigorous reporting methods, further confounds this.^20^ In addition, the trial of Smith et al. was noted to be underpowered. Due to the COVID‐19 pandemic, the authors were unable to recruit adequate numbers.^22^

Estrogen receptors have been identified throughout the female urogenital tract. It is typically the beta subtype of the estrogen receptor that is found most abundantly, especially in the urothelial tissues of the bladder. The findings by Bochenska et al. of multiple estrogen receptor subtypes (ESR1, ESR2, ESRα, and ESRβ) in the granulation tissue samples suggests that these tissues remain estrogen receptive throughout the postpartum period. In this study, the alpha estrogen receptor was much more abundant, especially in those who received local vaginal estrogen therapy. This may have to do with the characteristics of the population of the small sample size in the treatment group (*n* = 5), or may be associated with pathology, given that all the included participants had symptomatic painful or friable granulation tissue which was managed with excision.^21^ This may suggest a role for the treatment of symptomatic granulation tissue with vaginal estrogens, but larger studies are required.

Akin to the increased markers of cellular proliferation observed with the use of topical vaginal estrogens in the studies by Luz.^20^ and Bochenska et al.,^21^ the proliferative effect of topical estrogens on keratinocytes has been demonstrated,^4^ with multiple studies demonstrating that estrogens can promote synthesis of collagen and the extracellular matrix, contributing to re‐epithelialization and therefore healing.^5^, ^12^, ^23^ A metanalysis by Vodegel et al. demonstrated that collagen synthesis and tissue strength and vascularization increased with the use of estrogens.^23^ Granulation tissue was found to increase after the use of vaginal estrogens, which may be as a result of increased cellular proliferation; however, both animal and human studies were included, as well as all forms of estrogen preparations, not only vaginal, which could impact on applicability. There may therefore be a role for vaginal estrogens in vaginal epithelial or perineal wound healing postpartum. Of note, studies have not found evidence of improved healing or reduction in prolapse recurrence rates following pelvic floor repair surgery when vaginal estrogens are used.^8^ The difference in patient demographics, more commonly postmenopausal women, risk factor profile, and nature of the vaginal injury may account for this. Further studies are needed to assess the place for vaginal estrogens in perineal wound healing.

In one study included in this systematic review, an improvement in VuAS score following a 12‐week course of vaginal estrogen cream was noted. No other secondary variables, including UDI‐6 and FSFI scores, were found to be statistically or clinically significant, perhaps indicating that although objective vaginal changes could be seen on examination, subjective improvement in bladder and sexual function was not experienced by women.^22^ This may be due to other comorbid factors, such as mechanical stretching of the vagina and pelvic floor following pregnancy and delivery. Despite this, this study also found that the FSFI score, a measure of fecal incontinence, was lower for both trial arms at 12 weeks compared to six weeks postpartum. This may suggest that, independent of the use of vaginal estrogens, physiological healing processes following delivery enable restoration of pelvic floor and/or bowel tissues over time, resulting in subjective improvement. This is further implied by no patients, in either group, reporting a perineal pain score of >1 at 12 weeks postpartum, again suggesting physiological healing and tissue restoration. More investigations are required to establish the mechanisms of such processes and to see if this is also applicable to tissues of the bladder and pelvic floor, symptoms of which can be considerably distressing for women.

Although limited outcome‐related benefits were found from the use of vaginal estrogens in the postpartum period, the application of a topical vaginal estrogen (in the form of a cream in all the included studies) appeared to be acceptable and good compliance was maintained. This was more so for the treatment than for the placebo group suggesting that patients wished to continue to experience the effects offered by the vaginal estrogens, as opposed to simply a lubricating or moisturizing effect offered by a placebo cream.^22^ In addition, a common concern is that the use of hormonal products postpartum can impact milk supply or pass on to the baby through breastmilk. Luz found no decrease in milk supply amongst the participants, although the method of determining this was not documented.^20^ Nilsson et al. found no increased estrogen levels in breastmilk following the use of vaginal estrogens and Bochenska et al. found no difference in serum estrogen, progesterone, or dehydroepiandrosterone (DHEA) levels between those that had been using vaginal estrogens and those who had not,^24^ allowing clinicians and patients to be reassured about the peripartum safety of vaginal estrogens.

Limitations of this systematic review must be considered. The lack of available studies meeting the inclusion criteria, all with small sample sizes, potentially limits the accuracy of the conclusions which can be drawn. Each of the three included studies has different outcomes and uses different vaginal estrogen cream doses, making comparisons challenging. Performing high‐quality studies on the use of topical vaginal estrogens in postpartum women would add to the current knowledge base, especially studies including only breastfeeding women: this is the subject‐group known to be hypo‐estrogenic and therefore likely to benefit most from the local re‐introduction of estrogens vaginally. Additionally, the study by Smith et al. included participants who had sustained second degree perineal tears and above, which may have impacted the results obtained. Studies including postpartum women with vaginal atrophy, bladder, bowel and sexual dysfunction alone may exclude possible confounding influences of perineal trauma in the outcomes experienced by women and may be of further clinical benefit when deciding on treatment options.

Although a thorough literature search, using multiple databases, was conducted to the best of our knowledge, there may be some studies which were missed, especially ongoing trials and non‐English language papers. The applicability of the findings to clinical practice may also be restricted given only one in vivo study was performed. Furthermore, only one qualitative acceptability study was performed, data of which is especially important when considering the additional physical, emotional, and time constraints experienced by many postpartum women.

## 5 CONCLUSION

Histopathological changes, such as increased markers of cellular proliferation, have been seen in both vaginal fluid and granulation tissue in postpartum women using topical vaginal estrogens, as well as potential positive improvements in postpartum vulval characteristics, but statistical significance is still lacking. More evidence is required to determine causality: are the observed changes due to vaginal estrogen use or physiological healing over time as tissues return to their pre‐pregnancy baseline? Topical vaginal estrogens appear to be safe and tolerable to women, including for use in breastfeeding, and are already readily available in clinical practice.

With only three studies eligible for inclusion in this review, including only one randomized controlled trial, high‐quality evidence regarding the use of topical vaginal estrogens in postpartum women is significantly lacking, especially in vivo trials looking at all aspects of pelvic floor dysfunction. Given the increasing understanding of both lay women and clinicians of the importance of perinatal pelvic floor health, as well as the recent drive to improve access to such services in the UK, future research should focus on longitudinal, cohort and randomized controlled studies, incorporating larger sample sizes, to investigate the use of vaginal estrogens for a wide range of postpartum pelvic floor pathology, including urinary and fecal incontinence.

## AUTHOR CONTRIBUTIONS

**Aysha Waheed:** Conducted the literature search and is the first author of the systematic review. **Annika Taithongchai:** Reviewed and edited the systematic review article. **Dudley Robinson:** Reviewed and edited the systematic review article.

## FUNDING INFORMATION

No funding was sought for the compiling or writing of this systematic review.

## CONFLICT OF INTEREST STATEMENT

No conflicts of interest from any of the listed authors.

## Supporting information


**Appendix S1.** Search term used to conduct the systematic review.

**Figure S2.** Flow diagram to illustrate the literature search used to conduct this systematic review.

**Table S3.** Data table for this systematic review.

## Data Availability

The data that supports the findings of this study are available in the Supplementary Material of this article.
