# Supplementary material for: The use of topical vaginal estrogens in postpartum women: A systematic review
Source: Acta Obstet Gynecol Scand. 2026 May 4;105(7):1405–10. doi: 10.1111/aogs.70241 (PMC13308989; doi:10.1111/aogs.70241)
Supplement: Supplementary file 1 — Appendix S1. Search term used to conduct the systematic review. [file AOGS-105-1405-s002.docx]

**Supporting Information - Appendix 1 – S1**

Search Term:

((vaginal) OR (topical) OR (intravaginal) OR (intra-vaginal) OR (per vaginum) OR (per vagina) OR (pessary) OR (cream) OR (gel) OR (ring) OR (medication) OR (dose) OR (drug)) AND ((estrogen) OR (oestrogen) OR (estriol) OR (vagifem) OR (ovestin) OR (oestriol)) AND ((post-partum) OR (postpartum) OR (post-natal) OR (postnatal) OR (post-delivery) OR (post-birth) OR (post-childbirth) OR (post delivery) OR (post birth) OR (post childbirth) OR (post child-birth) OR (post-child-birth) OR (puerperium) OR (post-labour) OR (post labour) OR (perineum) OR (perineal) OR (perineal injury) OR (perineal trauma) OR (perineal tear) OR (OASI) OR (obstetric anal sphincter injury)).
