# Supplementary material for: The use of topical vaginal estrogens in postpartum women: A systematic review
Source: Acta Obstet Gynecol Scand. 2026 May 4;105(7):1405–10. doi: 10.1111/aogs.70241 (PMC13308989; doi:10.1111/aogs.70241)
Supplement: Supplementary file 2 — Figure S2. Flow diagram to illustrate the literature search used to conduct this systematic review. [file AOGS-105-1405-s003.docx]

**Supporting Information Figure – S2**

**Records identified through PubMed database searching matching inclusion criteria**

**n = 7**

**Additional records identified through other sources (Medline, Embase, Internet Searching, Citation Tracking)**

**n = 2**

**Records excluded with reasons**

**n = 6**

- **Duplicate n = 2**
- **Unpublished trial (trial registration abstract only)**

**n = 4**

**Full-text articles screened**

**n = 9**

**Articles included in analysis**

**n = 3**
