# Supplementary material for: The use of topical vaginal estrogens in postpartum women: A systematic review
Source: Acta Obstet Gynecol Scand. 2026 May 4;105(7):1405–10. doi: 10.1111/aogs.70241 (PMC13308989; doi:10.1111/aogs.70241)
Supplement: Supplementary file 3 — Table S3. Data table for this systematic review. [file AOGS-105-1405-s001.docx]

**Supporting Information Table 1 – S3**

| Author | Sample | Study design | Vaginal estrogen | Aim | Outcome | Comparator | Side effects | Findings | Additional comments |
| --- | --- | --- | --- | --- | --- | --- | --- | --- | --- |
| Luz (1960) | **n = 10**  -‘normal’ post-partum women receiving vaginal estrogens  -Mean age: Not disclosed  -Breastfeeding women n =10  -Perineal trauma: Not disclosed | Prospective | 0.1mg dienestrol per cubic centimeter (cc)  **Dose**: 5 cc dose daily until D15 postpartum | Evaluate the potential ability of puerperal epithelium to react to local estrogens | -Vaginal fluid for vaginal smear sample daily during hospital stay, between D11-15 and 5-7 days after stopping vaginal estrogens | Vaginal smears taken from ‘normal postpartum smears’ from those not taking vaginal estrogens | Nil | - No reduction in milk production found  - Proliferative cell-type pattern D3-4 (cornification and karyopyknosis)  -After D4 polygonal intermediate and superficial basophilic squamous cells dominated  -Fewer inflammatory cells in those using vaginal estrogens (absent after D4)  -D11-15 strong proliferative reaction – high cornification and karyopyknosis)  -After stopping vaginal estrogens: rapid vaginal involution + sub-atrophic smear, marked reduction in cornification and karyopyknosis | -Small sample size  -Minimal sample demographic information  -No information on demographics of comparator population samples were obtained from  -No comment on randomization or selection criteria  -No comment on perineal trauma / postpartum symptoms or pathology – only ‘normal’ women included (without definition)  - Data only available up to max. D22 postpartum |
| Bochenska et al. (2021) | **n = 16**;  -Women referred to a peripartum clinic with symptomatic perineal or vaginal granulation tissue (caused pain or bleeding) and required tissue removal  -5 post-partum women had received vaginal estrogens  -11 post-partum controls  -Mean age: 32.8 yrs  -Breastfeeding women n=12  -Perineal trauma: n=16 – 56% 3rd degree tear; 6% 4^th^ degree tear | Prospective | Topical vaginal estradiol (E2) cream  **Dose**: dose, method and area of application not standardized, frequency ranged from nightly to twice per week | To evaluate the biomolecular properties of vaginal and perineal granulation tissue in postpartum women and assess the potential impact of vaginal estrogen application | -RNA / PCR / histology / immunohistochemistry on vaginal or perineal granulation tissue excised from symptomatic postpartum women | Granulation tissue samples removed from symptomatic women who were not given vaginal estrogens | Not documented | -Granulation tissue most commonly in the midline (69%) and lateral vagina (25%)  -Granulation tissue formed of glandular and stromal tissues  -No H&E staining / structural changes between granulation tissue between groups  -Similar ESR1 and ESR2 estrogen receptor expression between the two groups  -No difference in serum estrogen / DHEA / progesterone levels between groups  -Alpha estrogen receptor predominant receptor in postpartum perineal/vaginal granulation tissue of both groups, but more abundant in estrogen-treated women (111xmore)  -No change in estrogen-positive inflammatory cytokines (TNF and IL-11) after estrogen use  -CyclinD1 (CCND1) a marker of cell proliferation increased after estrogen use (not statistically significant), but not seen in Ki67 another marker of cell proliferation | -Pilot study  -Small sample size  -Unequal trial arms  -No ethnicity data of trial participants  -Demographic information available  - No standardization of vaginal estrogen dose, duration of use, method and area of application  -Not all samples underwent all testing due to small sizes of the granulation tissue samples  -vaginal and perineal granulation contains estrogen receptors which could be targeted in treatment  -Suggestion of vaginal estrogens resulting in cellular proliferation and gene up-regulation -lack of statistically significant data |
| Smith et al. (2022) | **n = 59**  -31 primiparous women who sustained a 2^nd^ degree perineal laceration or above following a term vaginal delivery vaginal estrogen cream  -28 controls received placebo vaginal estrogen cream  -Mean age: 28.6 years  -Breastfeeding women: 46% at 12 weeks  -Perineal laceration: 12% had a 3^rd^ or 4^th^ degree tear | Randomized placebo-controlled trial | 0.01% Mylan estradiol cream  **Dose:** 1g twice weekly for 12 weeks  OR g Versabase placebo cream or 12 weeks | To evaluate the efficacy, acceptability and safety of local estradiol for treating genitourinary symptoms in primiparous women with perineal lacerations following a term vaginal delivery | -Vulvovaginal symptoms at 12 weeks postpartum  -Perineal pain  -Quality of life  -Sexual function  -Ease of use  -Likelihood of continued use  -Adverse events  -Vulvar Assessment Scale (VuAS)  -Vaginal Health Assessment (VHA)  -Edinburgh Postnatal Depression scale (EPDS)  -Urinary Distress Inventory-6 (UDI-6)  -Faecal Incontinence Severity Index (FISI)  -Female Sexual Function Index (FSFI) | Primiparous women who sustained a perineal laceration following a term delivery randomized to receive vaginal estrogen placebo | -1xUTI in placebo arm – deemed not relevant to the study | - -0.10 improvement in Vulvar Assessment Scale score after 12 weeks of using vaginal estrogens (50% reduction from 0.20 in placebo arm and 0.10 in treatment arm)  -No improvement seen in any other measures in the vaginal estrogen group  -Acceptable and safe for women  -No difference in VuAS and VHA vaginal assessments at 12 weeks between groups  -Mean FSFI lower at 6/52 than 12 weeks and 6 months ?improves over time with or without intervention  -No patients recorded perineal pain score >1 on NRS scale at 12 weeks ?improved with time  -52% very satisfied with treatment in estradiol arm at 12/52; only 33% of placebo arm  -58% used all the time in estradiol arm  -52% reported being very much better in the estradiol arm | -Pilot study  -Randomized placebo-controlled trial design  -Intention to treat analysis  -76% non-Hispanic white  -94.9% completed 6 and 12 week follow-up  -89.8% completed 6 month follow-up  -Poor enrolment resulted in the trial being inadequately powered |
